# Illustration of association between change in prostate-specific antigen (PSA) values and time to tumor status after treatment for prostate cancer patients: a joint modelling approach

**DOI:** 10.1186/s12894-023-01374-8

**Published:** 2023-12-06

**Authors:** Madiha Liaqat, Shahid Kamal, Florian Fischer

**Affiliations:** 1 College of Statistical and Actuarial Sciences (CSAS), University of the Punjab, Lahore, Pakistan; 2 Institute of Public Health, Charité – Universitätsmedizin Berlin, Berlin, Germany

**Keywords:** Prostate cancer, Prostate-specific antigen, PSA, Time to Tumor status, Joint modelling, Dynamic prediction

## Abstract

**Background:**

Prostate cancer (PCa) is the most prevalent tumor in men, and Prostate-Specific Antigen (PSA) serves as the primary marker for diagnosis, recurrence, and disease-free status. PSA levels post-treatment guide physicians in gauging disease progression and tumor status (low or high). Clinical follow-up relies on monitoring PSA over time, forming the basis for dynamic prediction. Our study proposes a joint model of longitudinal PSA and time to tumor shrinkage, incorporating baseline variables. The research aims to assess tumor status post-treatment for dynamic prediction, utilizing joint assessment of PSA measurements and time to tumor status.

**Methods:**

We propose a joint model for longitudinal PSA and time to tumor shrinkage, taking into account baseline BMI and post-treatment factors, including external beam radiation therapy (EBRT), androgen deprivation therapy (ADT), prostatectomy, and various combinations of these interventions. The model employs a mixed-effect sub-model for longitudinal PSA and an event time sub-model for tumor shrinkage.

**Results:**

Results emphasize the significance of baseline factors in understanding the relationship between PSA trajectories and tumor status. Patients with low tumor status consistently exhibit low PSA values, decreasing exponentially within one month post-treatment. The correlation between PSA levels and tumor shrinkage is evident, with the considered factors proving to be significant in both sub-models.

**Conclusions:**

Compared to other treatment options, ADT is the most effective in achieving a low tumor status, as evidenced by a decrease in PSA levels after months of treatment. Patients with an increased BMI were more likely to attain a low tumor status. The research enhances dynamic prediction for PCa patients, utilizing joint analysis of PSA and time to tumor shrinkage post-treatment. The developed model facilitates more effective and personalized decision-making in PCa care.

## Background

Prostate Cancer (PCa) is a major cause of cancer-related issues in men. According to Siegel et al. [1], almost 1.4 million new cases are registered each year worldwide. Diagnosis of PCa is a challenging task due to the involvement of many risk factors and biomarkers [2]. Treatment options include external beam radiotherapy (EBRT), androgen deprivation therapy (ADT), prostatectomy, and combinations thereof, chosen based on the severity of cancer [3]. EBRT and ADT in combination are the most appropriate treatments for moderate to higher-risk cancer [4]. Patients are monitored during and after treatment to get disease insights for PSA measurements [5], which is a serine protease protein biomarker discharged by prostate [6]. Sheikh et al. [7] investigated the association between longitudinal PSA and survival outcomes to explore the time-to-low or high-grade PCa.

Interest in personalized medicine has been increasing in recent years in the biomedical fields of research, and physicians are accustomed to customizing treatment decisions by monitoring biomarkers of disease progression to improve medical care and patients’ well-being. For example, monitoring a patient’s PSA level allows clinicians to make predictions about the recurrence of PCa after treatment [8]. However, a separate analysis of longitudinal biomarker data and event time outcomes may produce misleading estimates by ignoring any possible dependence structure between both outcomes. Joint modelling of longitudinal and event time data is preferred in this case as compared to separate analyses to fully utilize the available information and obtain unbiased results [9]. Joint modelling is a versatile approach for deriving event time probabilities to forecast future events, considering various associated structures for longitudinal and event time processes, thereby providing better predictions about individuals [10].

The joint modelling strategy for dynamic prediction utilizes joint information on tumor shrinkage and PSA measurements, allowing it to make updated predictions for PCa patients, aiming to provide patient-specific trajectories of PCa progression and time-to-event (TTE) data. For this purpose, longitudinal biomarker data are collected, with the primary outcome consisting of the time until the occurrence of a pre-specified event. Sometimes, multiple longitudinal outcomes of different types are collected [11, 12], which may have an association with TTE outcomes to discover inherent characteristics of patients and gain insights into disease progression. It is also interesting for researchers to obtain subject-specific predictions for one of the outcomes, whether longitudinal or event time [10, 13].

The framework of individual dynamic predictions is based on available information related to future events. Dynamic prediction models support medical decision-making, where changes in covariates modify over time to predict an event occurring in the future. Changes over time update the prognosis, accounting for changes in biomarkers and patients’ characteristics. All appropriate changes must be included in study variables to optimally assess prognosis. An optimal prediction model is employed to intuitively predict future outcomes, facilitating patient-informed decision-making [14].

Quantifying the risk of an event related to disease progression at the individual level is facilitated by information collected at the diagnosis stage and during follow-up visits [15]. Ferrer et al. [16] compared the accuracy of survival predictions between joint and landmark modelling. Maziarz et al. [17] applied conditional survival models to obtain predictions and concluded that conditional models exhibit better computational efficiency in prediction compared to joint models. Single and multiple markers have been employed to enhance the prediction of future events [18].

Based on already reported biomarkers, most studies considered PSA to be overdiagnosed at a rate ranging from 1.7 to 67% [19]. This research aims to assess the tumor status (low or high) of PCa patients’ post-treatment (utilizing EBRT, ADT, prostatectomy, and combinations) with a PSA level of ≥ 4 ng/ml. It proposes the best-fitted joint model to illustrate any association between longitudinal PSA and the time to tumor status. In addition, the proposed joint model is used to dynamically predict tumor status within a fixed time for a subject that is still at risk before time t [10]. Many authors have studied future event probabilities for individuals based on joint modelling of longitudinal measurements, event time outcomes, and other covariates [20].

This article develops a dynamic prediction model, utilizing longitudinally collected PSA to predict the future tumor status for PCa patients after treatment. The best longitudinal sub-model and event time sub-model are jointly specified for prediction purposes, in such a way that longitudinally assessed continuous PSA is utilized as an event time predictor.

## Materials and methods

### Study data

Current study focuses on 1504 men with primary PCa and treated by EBRT, ADT, prostatectomy, or a combination of these interventions. The dataset for this study was obtained from a renowned cancer hospital in Pakistan. Ethical approval was secured from the departmental head and dean of sciences at the University of the Punjab, with permission granted by the hospital authority. Patients registered at the hospital between 2012 and 2019, diagnosed with PCa, and followed for at least two visits were included in the study. The data were manually entered into Excel sheets from patients’ files, ensuring completeness and accuracy while excluding any insufficient information. During follow-up, treatment efficacy is assessed by observing the patients’ tumor status, detecting local or distant recurrence, and noting instances of death, whether attributed to PCa or not. Specifically, our focus is on the time taken for tumor shrinkage, a parameter monitored by physicians through regular visits and observing PSA measurements after the initial treatment. PSA measurements were collected between the end of initial treatment and the occurrence of study event, which is tumor shrinkage up to satisfactory level or event not happened at the end of study period. As shown in Table 1, this study observed post-treatment PSA levels in the log scale (logPSA), BMI (kg/m2), time to tumor shrinkage (in months), and treatment (categorized into 7 groups: 2, 3, 4, 5, 6 versus 1 = ADT as a reference) variables.

**Table 1:** Baseline characteristics

	Right Censored (*N* = 544)	Low (*N* = 960)	Overall (*N* = 1504)
**PSA**			
Mean (SD)	40.5 (17.3)	29.3 (14.7)	33.4 (16.6)
Median [Min, Max]	41.8 [3.35, 129]	30.1 [0.280, 89.7]	33.7 [0.280, 129]
**BMI**			
Mean (SD)	19.1 (2.27)	19.7 (2.04)	19.5 (2.14)
Median [Min, Max]	19.2 [14.0, 26.5]	19.8 [14.1, 26.0]	19.6 [14.0, 26.5]
**Treatment**			
ADT	108 (19.9%)	220 (22.9%)	328 (21.8%)
ADT + EBRT	168 (30.9%)	185 (19.3%)	353 (23.5%)
ADT + Prostatectomy	49 (9.0%)	29 (3.0%)	78 (5.2%)
ADT + Prostatectomy + EBRT	16 (2.9%)	11 (1.1%)	27 (1.8%)
EBRT	101 (18.6%)	377 (39.3%)	478 (31.8%)
Prostatectomy	83 (15.3%)	78 (8.1%)	161 (10.7%)
Prostatectomy + EBRT	19 (3.5%)	60 (6.3%)	79 (5.3%)
**Time**			
Mean (SD)	224 (83.9)	250 (90.1)	241 (88.8)
Median [Min, Max]	218 [64.0, 458]	248 [68.0, 486]	237 [64.0, 486]

**Fig. 1:**
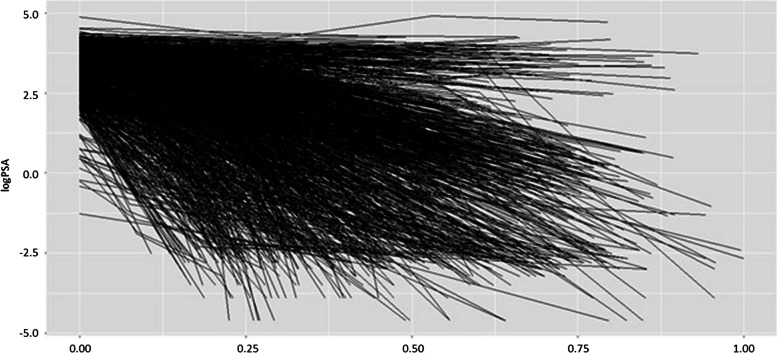
Individual and mean profiles of observed PSA data over time

Repeated PSA measurements are taken during check-ups; its increased level after treatment indicates the growth of cancer cells. The PSA individual trajectories collected between the end of initial treatment and the occurrence of the event are depicted in Fig. 1. In general, this longitudinal process highlights variations in the biomarker’s long-term changes, reflecting both “tumor shrinkage” and instances of “censorship”, for example, based on study aim which is to illustrate potential relationship between PSA and time until patients achieve a satisfactory tumor status as directed by physicians.

The time to tumor shrinkage represents the outcome variable categorized as ‘Yes’ for patients with the event of interest or ‘No’ for those patients who did not experience tumor shrinkage or left the follow-up study. ‘Yes’ is coded as 1 and ‘No’ is coded as 0.

Considering the impact of PSA levels on PCa patients’ recovery, utilizing a statistical model is crucial for understanding the relationship between PSA measurements and tumor status. The dynamic progression of PCa varies among patients, highlighting the PSA biomarker’s significance in describing disease progression and its’ correlation with tumor status. This potential is unveiled through the combined analysis of repeated PSA measurements and time to tumor shrinkage variables. Table 1 illustrates the baseline characteristics of PCa patients. The median follow-up number of times is 3 per patient with a range of 1 to 5, which are distributed unequally among individuals. Two outcomes are distributed as $log PSA \left(1.96\pm 2.03\right),$ and shrinkage of tumor (Yes, No). The event of interest for this study is individuals’ condition (1: tumor shrinkage, 0: right censored) at the end of follow-up time, from 1,504 patients 960 observed events of interest, and 544 were right censored.

### Statistical modelling and analysis

A joint model [21, 22], incorporating mixed-effects and event time components, has been developed to capture the relationship between PSA and tumor status. The mixed-effects model describes the evolution of PSA over time, taking into account both fixed and random effects. The event time sub-model, employing the Cox proportional hazards model, analyzes censored data, with baseline predictors including BMI and treatment.

A simple mixed-effects model for longitudinal data with general form is written as,

1 $${y}_{i}\left(t\right)={u}_{i}\left(t\right)+{\varepsilon }_{i}\left(t\right),$$

where, ${u}_{i}\left(t\right)$ is mean of predictor for both fixed and random effects, ${\varepsilon }_{i}\left(t\right)$ is an error term. Investigation of the effect of covariates on repeated PSA measurements can be applied using quadratic, cubic, or non-parametric fits.

**Fig. 2:**
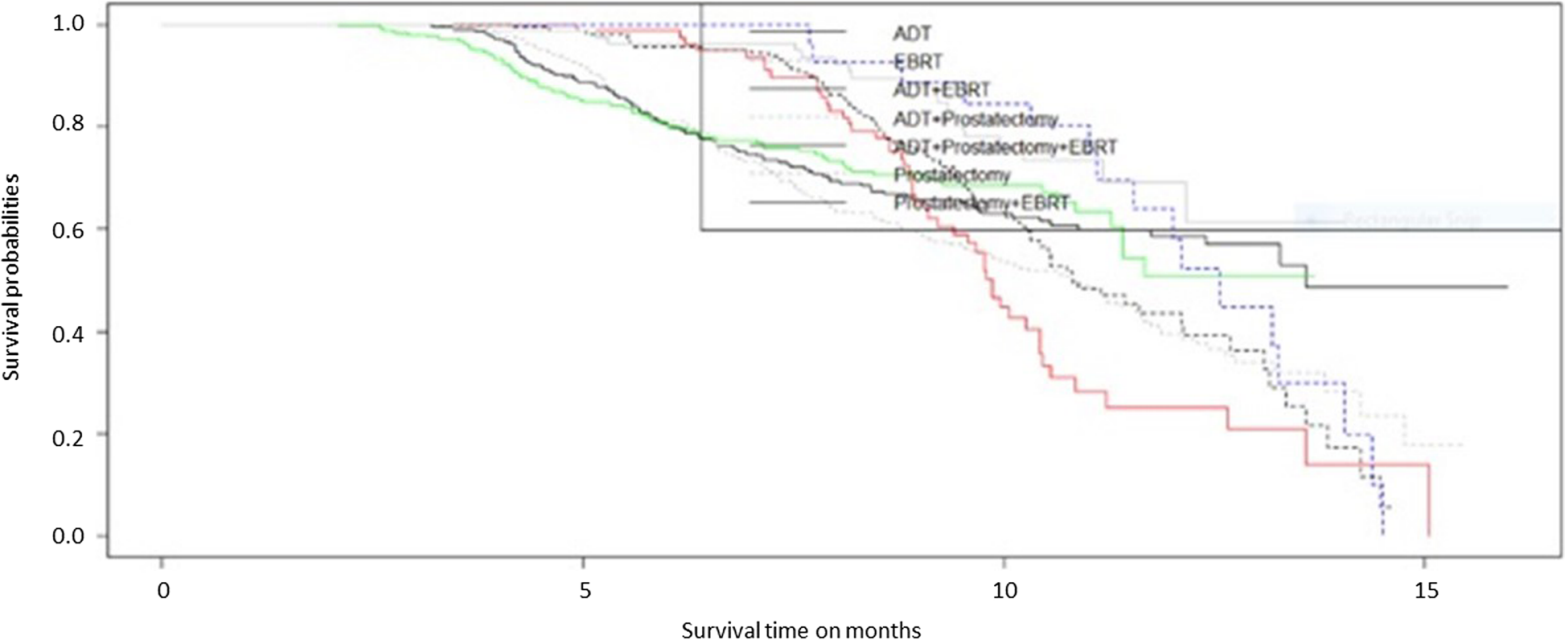
Kaplan-Meier estimates of the probability of survival for individuals on each treatment

An event time sub-model is formulated for censored data, as not all patients experienced an event of interest. Mostly right censoring occurs, due to dropouts before the end of follow-up time [23]. Cox proportional hazards (PH) [24] is the most popular semi-parametric model to analyze event time data [25], which is formulated as,

2 $${\pi }_{i}\left(t\right)={\pi }_{0}\left(t\right)\text{exp}\left({\gamma }^{T}{\omega }_{i}\right),$$

where ${\pi }_{0}\left(t\right)$ is a baseline hazard function at time $t$, ${\omega }_{i}$ denotes baseline predictors for regression coefficients’ vector $\gamma .$ The Kaplan-Meier event curve in Fig. 2 illustrates the time to low-status tumor following treatment combinations. It indicates an increased probability of time to low tumor with the administered treatments, as compared to other treatments ADT observed more effective in terms of time to low tumor status. The baseline hazard can either be unspecified or parametrically modeled using appropriate distributions such as Weibull, Gamma, Exponential, and others [26].

The joint model specifies the hazard of the event, which is dependent on individual characteristics of its longitudinal trajectory, as follows

3 $${\pi }_{i}\left(t\right)={lim}_{\varDelta t\to 0}\frac{\text{Pr}\left\{t\le {T}_{i}^{*}+\varDelta t|{T}_{i}^{*}\ge t,{\mathcal{M}}_{i}\left(t\right),{\omega }_{i}\right\}}{\varDelta t},$$

where, ${\mathcal{M}}_{i}\left(t\right)=\{{\mu }_{i}\left(s\right),0\le s<t\}$ is a history of unobserved longitudinal process ${\mu }_{i}\left(s\right)$ up to time t, and ${\omega }_{i}$ is a vector of time-varying covariates.

Constructing a joint model involves integrating various association structures to unify longitudinal and event time processes. Commonly used association structures include current value, shared random effects, and current value and slope [27]. The current value association structure assumes that the true value ${\mu }_{i}\left(t\right)$ of longitudinal measure at time $t$ is predictive of the risk of experiencing an event at the same time. The Cox’s PH sub-model with this association structure is written as,

4 $${\pi }_{i}\left(t\right)={\pi }_{0}\left(t\right)\text{exp}\left({\gamma }^{T}{\omega }_{i}+{\alpha }_{1}{\mu }_{i}\left(t\right)\right),$$

$\alpha$ is a vector of associated parameters to quantify the association between longitudinal process and hazard for the event at time $t$. It is interpreted as one unit increase in current value is associated with $\text{e}\text{x}\text{p}\left({\alpha }_{1}\right)$ increase in risk of event at the same time, given that event has not occurred before $t.$

In a shared random effects association structure, random effects from the longitudinal sub-model are incorporated into the relative risk sub-model as linear predictors, facilitating the sharing of random effects between the two [28]. Another association structure, current value and slope, establishes a linkage between event time and longitudinal sub-models by adding the rate of change of measurement at time $t$ estimated by taking the derivative of ${\mu }_{i}\left(t\right)$ with respect to time. A sensitivity analysis is conducted to select the appropriate association structure. The structure is chosen based on the BIC criteria, opting for the model with the lowest BIC value.

For dynamic prediction, the three-step process is employed to develop a prediction model that accounts for both baseline patient characteristics and longitudinal measurements of PSA values.

### Joint PSA and time to tumor status model

We utilized a series of joint logPSA and time-to-tumor-status models, exploring diverse options for both longitudinal and event time sub-models, along with various association structures for joint modelling. The first step describes the evolution of PSA measurements over time, and the second step utilizes this information to model event time. Finally, dynamic prediction is performed using the proposed joint model. As a preliminary step, a covariate selection process is carried out for a sub-model of the longitudinal outcome, and heterogeneity in residual plots is mitigated using a logarithmic scale of PSA.

A mixed-effects model is proposed for the evolution of PSA over time to account for the positive correlation between observed measurements within the same patient. This model includes time (in months) and baseline treatment variables (ADT, prostatectomy, EBRT, and combinations of these). Based on BIC [29] criteria, the best model for logPSA repeated measures is formulated as,

5 $${y}_{ij}=({\beta }_{0}+{b}_{0i})+{(\beta }_{1}+{b}_{1i}){Months}_{i}+{\beta }_{2}{Months}_{i}^{2}+{\beta }_{3}{Treatment}_{i}+{\beta }_{4}{Treatment}_{i}\times {Months}_{i}+{\beta }_{5}{Treatment}_{i}\times {Months}_{i}^{2}+{\varepsilon}_{i}\left(t\right)$$

In the event time process, following the initial covariate selection, treatment and BMI are identified as significant covariates.

6 $${\pi }_{i}\left(t\right)={\pi }_{0}\left(t\right)\text{e}\text{x}\text{p}({\beta }_{1}{BMI}_{i}+{\beta }_{2}{Treatment}_{i}),$$

Joint model prediction based on event time probabilities and future PSA observations for any new patient $j$, utilizing longitudinal PSA values ${\mathcal{M}}_{i}\left(t\right)=\{{\mu }_{i}\left(s\right),0\le s<t\}$ and baseline covariates ${\omega }_{j}.$ Conditional probability ${\pi }_{j}\left(u\right|t)$ is used to predict about patient $j$, who will have low tumor for time $u>t$ observing PSA. Using information available at time $t$, prediction is updated dynamically at any follow-up visit time ${t}^{{\prime }},$ such that ${t}^{{\prime }}=t<{t}^{{\prime }}<u$ to produce a new prediction ${\omega }_{j}\left(u|{t}^{{\prime }}\right)$.

## Results

Joint modelling approach for dynamic prediction of tumor progression and shrinkage allows individual predictions to be made for PCa patients, based on PSA measurements after treatment. This study aims to provide patient-specific trajectories of PCa progression and TTE. Different forms of association that relate longitudinal PSA with time-to-tumor status are assessed, and predictions are averaged over different models via Bayesian model averaging. Individual follow-up time ranged from 64 to 486 days, with a median of 237 days. Estimates of associated structure parameters are considered in two to three models, which indicated that PCa tumor shrinkage at any time point is associated with the rate of change in PSA level.

The model having the interaction effect of treatment with degree-2 polynomial of months best fits the data, on the basis of low BIC. To make sure about model fitting for longitudinal data, ${R}^{2}$ criteria were applied [30]. Marginal ${R}^{2}$ explained the proportion of variance in fixed effects, and conditional ${R}^{2}$ explained the proportion of variance in fixed and random effects. In our case, the proportion of variance explained in the chosen longitudinal model is 90%; therefore, this final sub-model is an excellent fit to pursue our joint modelling estimation procedure.

The Cox PH sub-model is employed for event time data with a nonparametric baseline hazard function, following an assessment of the PH assumption using the Schoenfeld Residuals Test [31].

B- and regression splines approaches can also be employed, with different numbers and positions of nodes, and criteria such as BIC are used to determine the best nodes [11].

 Joint model applied to explore the association between longitudinal trajectories in PSA and tumor status after treatment is presented in Table 2, including parameter estimates and 95% CI with the current value association structure. Variance-covariance matrix for the current value association structure is $$\left[\begin{array}{c} 0.574\\ -0.265\end{array} \begin{array}{c}-0.265\\ 0.412\end{array}\right]$$, with a 0.708 residual value. Results depict that follow-up time has a significant negative effect on PSA levels, with increasing months in treatment associated with decreases in PSA levels. Associated parameters show that PSA and time to tumor status both are associated positively, by increasing one unit in the current value of longitudinal PSA, the chances of having low tumor increases with 0.368 units at the same time, given that shrinkage of the tumor did not occur before that particular time. It is also assumed that this association rate is the same across individuals. In terms of treatment options, ADT has a positive significant effect on PSA levels as compared to other treatment combinations. Months^2^ has a negative significant effect on decreasing PSA level with an average rate 0.019. Months with all treatment combinations have a significant effect on patients’ PSA measurements. ADT + EBRT, Prostatectomy + EBRT, and EBRT with second-degree polynomial of follow-up time (months) interactions have a negative significant effect on PSA with average change. The event time sub-model has a significant predictor of BMI, by increasing one unit in BMI, patients have a good chance of having a good status on average 0.119. Treatment combinations ADT + Prostatectomy, ADT + Prostatectomy + EBRT, EBRT, Prostatectomy, and Prostatectomy + EBRT, significantly have affected having good tumor status as an event of interest with time.

**Table 2:** Parameter estimates and 95% CI of joint model with current value association structure

**Parameter**	**Value**	**2.5%**	**97.5%**
Longitudinal Process			
**Intercept**	3.641	3.567	3.717
**Months**	-0.643	-0.700	-0.587
**Treatment**			
ADT+EBRT	0.180	0.077	0.285
ADT+Prostatectomy	0.232	0.050	0.417
ADT+Prostatectomy+EBRT	0.378	0.094	0.655
EBRT	-0.978	-1.078	-0.881
Prostatectomy	0.067	-0.072	0.202
Prostatectomy+EBRT	-0.267	-0.518	-0.032
**I(Month**^**2**^**)**	-0.019	-0.024	-0.013
**Months:Treatment**			
Months:ADT+EBRT	0.270	0.190	0.350
Months:ADT+Prostatectomy	0.428	0.302	0.554
Months:ADT+Prostatectomy+EBRT	0.538	0.341	0.730
Months:EBRT	-0.424	-0.496	-0.350
Months:Prostatectomy	0.278	0.187	0.372
Months:Prostatectomy+EBRT	0.423	0.292	0.556
**Treatment:I(Months**^**2**^**)**			
ADT+EBRT:I(Months^2^)	-0.009	-0.017	-0.001
ADT+Prostatectomy:I(Months^2^)	-0.001	-0.011	0.009
ADT+Prostatectomy+EBRT:I(Months^2^)	0.002	-0.011	0.016
EBRT:I(Months^2^)	0.038	0.029	0.047
Prostatectomy:I(Months^2^)	0.008	-0.001	0.017
Prostatectomy+EBRT:I(Months^2^)	-0.017	-0.030	-0.049
Event Time Process			
**BMI**	0.119	0.028	0.204
**Treatment**			
ADT+EBRT	-0.192	-0.467	0.093
ADT+Prostatectomy	-0.486	-0.902	-0.100
ADT+Prostatectomy+EBRT	-1.329	-1.930	-0.764
EBRT	0.721	0.427	1.025
Prostatectomy	-0.468	-0.783	-0.132
Prostatectomy+EBRT	-1.023	-1.534	-0.539
**Assoct**	0.368	0.309	0.426
**tauBs**	1.827	0.622	4.318

**Fig. 3:**
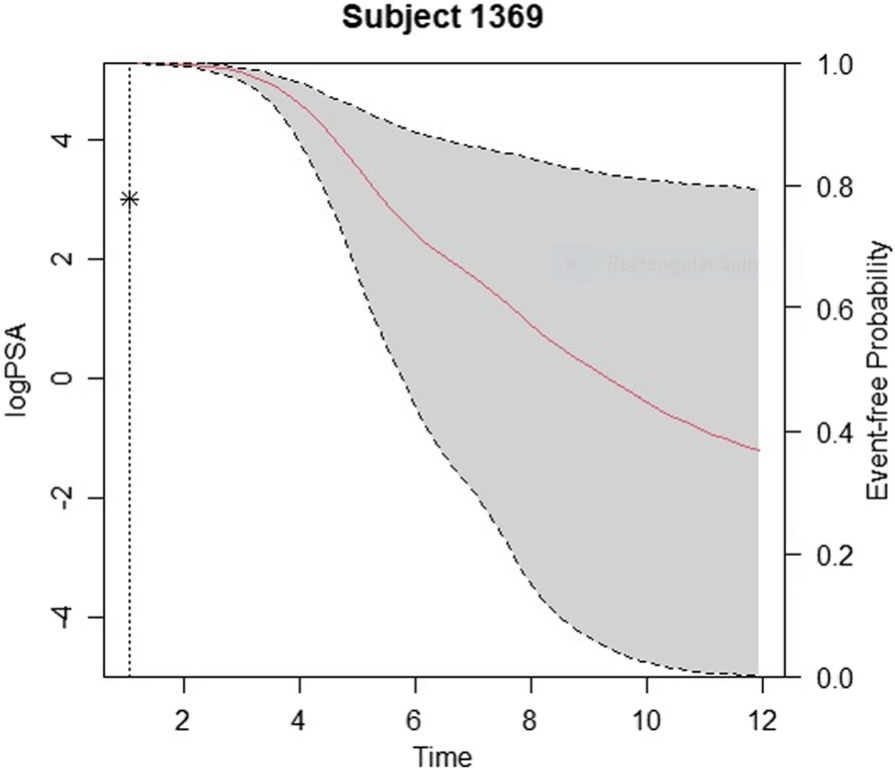
Observed (star) and estimated (red line) PSA till time of last observed value; and event time probability predicted at time of last observed PSA for patient 1369

In joint modelling for dynamic prediction, the choice of predictors is theoretically integrated into both sub-models. The longitudinal sub-model aims to describe the trajectory of a variable over time for each individual, considering measurement noise, while the event time model forecasts the tumor shrinkage after treatment. Any predictor that enhances the prediction accuracy of both sub-models can be included. Joint modelling is employed for dynamic predictions of events at any future time based on the information available up to a specific time point, denoted as t > 0 [32]. Figure 3 illustrates the future prediction for patient 1369 based on the last observed PSA value in month 0.912.

We evaluate the discrimination of the model for patients who observed the event and those who did not by using the time-dependent estimated Area Under the Curve (AUC). According to AUC criteria, values near 0.5 indicate random chance, while values closer to 1 indicate better model discrimination [33]. The time-dependent AUC for our proposed joint model is 0.8568 at 18 months, utilizing information up to 15 months, with 8 subjects still at risk.

## Discussion

Our results support the hypothesis of an association between PSA measurements and time to tumor status. We found that the time to prostate low tumor status increases with lower level of PSA and a faster change in rate of low tumor status incorporating initial treatment. Only treatment, and time are statistical significance as predictors of PSA measurements, while baseline BMI and treatment were identified as predictors of lower tumor status for prostate cancer patients.

This article also demonstrates the utilization of joint modelling to predict tumor status using longitudinal and event time data, specifically focusing on PSA measurements post-treatment. Applying models that consider all available information over time enhances individual prognostication. We estimated the joint modelling of longitudinal PSA with time and time to tumor status after treatment, incorporating a model that accounts for measurement error due to time-dependent covariate endogenous effects and non-random dropout. The current value association structure was employed to predict tumor status for PCa patients’ post-treatment by combining baseline covariates and longitudinal PSA.

Results indicate the model’s ability to predict tumor progression based on PSA post-treatment. The chosen model includes a quadratic trajectory for PSA with significant associations between follow-up time, treatment, and PSA levels. The event time sub-model highlights the impact of BMI and treatment on the likelihood of tumor shrinkage. The results align with existing literature and clinical knowledge, highlighting a strong association between observed longitudinal PSA and PCa tumor status post-treatment. Notably, our proposed model suggests a substantial effect of PSA values in conjunction with treatment over the course of time. Our contribution lies in employing joint modelling techniques to efficiently estimate predictors for PSA trajectories that impact the time to PCa tumor status. The estimation of low PCa tumor status, based on the complete historical record of PSA evaluations for each patient, adds significant value.

While our research serves as an initial step, there are limitations. First, it focuses solely on the PSA biomarker, and future studies should explore associations among multiple biomarkers. Second, the model includes limited covariates, and incorporating additional time-varying covariates could enhance predictive ability.

The study underscores the significance of joint modelling in predicting tumor status based on PSA. The proposed model’s capacity to integrate both baseline characteristics and longitudinal PSA data enhances individual prognostication. Future endeavors will delve into the influence of additional longitudinal biomarkers and time-varying covariates, expanding upon the groundwork established in this study [34, 35]. Di Minno et al. [36] discussed the significance of 8-hydroxy-2-deoxyguanosine (8-OHdG) and 8-iso-prostaglandin F2α (8-IsoF2α) biomarkers in evaluating radicality and potential local recurrence after PCa surgery. Our future plan involves illustrating the joint evaluation of 8-OHdG, 8-Iso-PGF2α and time to local recurrence, after different treatment options, and exploring possible association structures to enhance dynamic prediction in a long-term follow-up study. This pursuit aims to refine predictive models and provide valuable insights for clinical decision-making regarding PCa treatment and risk prediction.

## Conclusions

The joint modelling strategy proves effective in analyzing the correlation between repeated PSA measurements and tumor status in PCa patients. It is well observed that PSA levels decrease in patients who received ADT, and among these patients, a low tumor status is associated with a one-unit increase in BMI, compared to patients who received other treatments and had a low BMI. While it might seem plausible to derive inferences about the association between PSA measurements and tumor status through linear mixed models for time to tumor status, it is crucial to note that these models can yield biased results in the presence of nonrandom dropout. Additionally, fitting such models requires calculating the time elapsed between each follow-up and low tumor status, limiting the analysis to individuals with known event times, potentially leading to a smaller and biased sample. The joint modeling approach employed here addresses these issues, specifically designed to mitigate biases from nonrandom dropout. Patients who continued follow-up until the end of the study period and those whose physicians were dissatisfied with their tumor status are treated as censored, ensuring their inclusion in the analyses and preventing exclusion biases.

The quality of our results hinged on selecting a fitting model for the data. While many studies typically employ likelihood information criteria for selecting joint models, our research highlights that preferences in terms of model fitting complexity and dynamic prediction can differ. We rigorously assessed potential models, ultimately choosing the best fit based on criteria, particularly those recommended for longitudinal and event time data. The exploration of different association structures enhances the model’s robustness. Future studies should explore multiple biomarkers and additional covariates to improve predictive models for PCa treatment and risk prediction.

## Data Availability

Data is available from corresponding author upon reasonable request.

## Abbreviations

ADT: Androgen deprivation therapy
BMI: Body-mass-index
EBRT: External beam radiotherapy
PCa: Prostate cancer
PSA: Prostate-specific antigen
TTE: Time-to-event
